# Supplementary material for: A Computational Journey Toward an Optimal Design for Metamaterial Epicardial Passive Sleeves
Source: Adv Healthc Mater. 2025 Oct 23;15(4):e01369. doi: 10.1002/adhm.202501369 (PMC12836452; doi:10.1002/adhm.202501369)
Supplement: Supplementary file 1 — Supporting Information [file ADHM-15-0-s001.pdf]

ADVANCED
HEALTHCARE
MATERIALS

Supporting Information

for *Adv. Healthcare Mater.*, DOI 10.1002/adhm.202501369

A Computational Journey Toward an Optimal Design for Metamaterial Epicardial Passive Sleeves

Vahid Naeini, Emilio A. Mendiola, Ahmad Rafsanjani, Fergal B. Coulter, Qian Xiang, Jianyi Zhang, Peter Vanderslice, Vahid Serpooshan and Reza Avazmohammadi**

Supporting Information

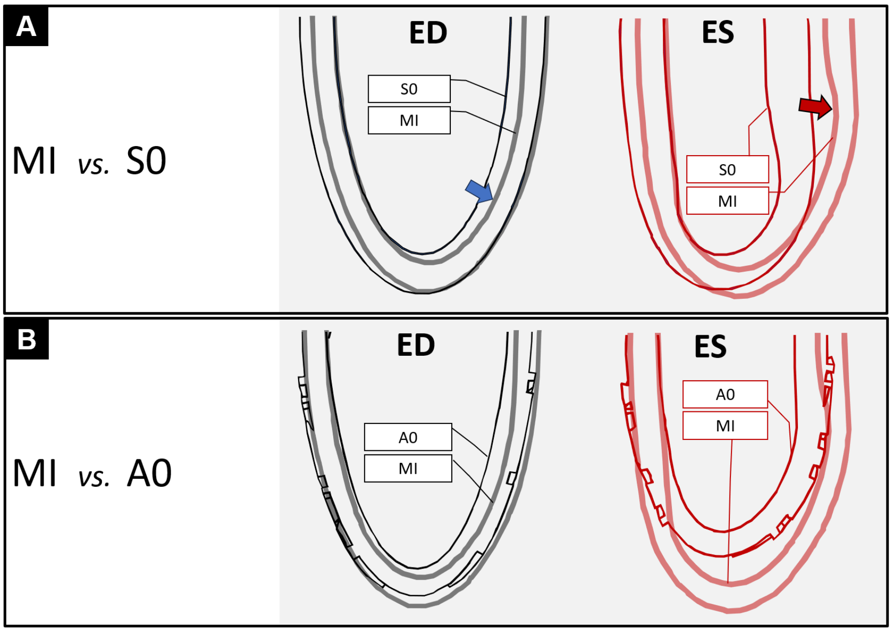

Figure S.1: ED and ES deformation comparisons:
(A) Comparison between the MI state and the simple continuum sleeve design, showing the deformations in ED and ES. Blue arrow shows the deformed wall in ED. The simple continuum sleeve reduces bulging at ES, indicated by the red arrow. **(B)** Comparison between the MI state and the auxetic sleeve design, showing the deformations in ED and ES.

Table S.1: A comparison of EDV, ESV, SV, and EF for various sleeve geometries. Changes in SV and EF are denoted as ΔSV and ΔEF, respectively. MI refers to the baseline post-infarct cardiac model without sleeve.

	**MI**	**S1**	**S2**	**S3**	**S4**	**S5**
θ	N/A	45°	45°	45°	45°	45°
ζ	N/A	1.0	1.0	1.0	1.0	1.0
EDV [mL]	0.6563	0.6135	0.6365	0.6244	0.6246	0.6226
ESV [mL]	0.5731	0.5370	0.5538	0.5433	0.5441	0.5423
SV [mL]	0.0832	0.0766	0.0827	0.0811	0.0805	0.0802
ΔSV	N/A	-8.0%	-0.6%	-2.6%	-3.3%	-3.6%
EF [%]	12.7	12.5	13.0	13.0	12.9	12.9
ΔEF	N/A	-1.6%	2.5%	2.4%	1.6%	1.6%

Table S.2: Functional parameters EDV, ESV, SV, and EF in the S1 continuum sleeve geometry with different fiber architecture orientations. MI refers to the baseline post-infarct cardiac model without sleeve.

	**MI**	**S1**	**S1**	**S1**
θ	N/A	0°	45°	90°
ζ	N/A	1.0	1.0	1.0
EDV [mL]	0.6563	0.6116	0.6135	0.6198
ESV [mL]	0.5731	0.5357	0.5370	0.5436
SV [mL]	0.0832	0.0760	0.0766	0.0763
ΔSV	N/A	-8.7%	-8.0%	-8.4%
EF [%]	12.7	12.4	12.5	12.3
ΔEF	N/A	-2.0%	-1.6%	-3.0%

Table S.3: Effect of varying the stiffness ratio ζ on EDV, ESV, SV, and EF at a constant fiber architecture angle θ of 90° in the S1 continuum sleeve geometry. MI refers to the baseline post-infarct cardiac model without sleeve.

	**MI**	**S1**	**S1**	**S1**	**S1**	**S1**
θ	N/A	90°	90°	90°	90°	90°
ζ	N/A	1.0	1.1	1.4	2.0	5.0
EDV [mL]	0.6563	0.6198	0.6191	0.6171	0.6074	0.5913
ESV [mL]	0.5731	0.5436	0.5428	0.5408	0.5379	0.5302
SV [mL]	0.0832	0.0763	0.0763	0.0763	0.0694	0.0610
ΔSV	N/A	-8.4%	-8.3%	-8.3%	-16.6%	-26.7%
EF [%]	12.7	12.3	12.3	12.4	11.4	10.3
ΔEF	N/A	-3.0%	-2.8%	-2.5%	-9.9%	-18.6%

Table S.4: Quantitative Analysis of Sleeve-Induced Rotational Mechanics

**Parameter**	**S4, 90°**	**S5, 90°**	**S5, 45°**
Torsion Metrics			
Peak Torsion (°)	5.22 ±2.44	4.27 ±2.54	6.74 ±3.22
End-Systolic Torsion (°)	3.77 ±2.44	-1.46 ±2.54	4.89 ±3.22
Torsion Range (°)	8.20	7.74	9.27
Coefficient of Variation (%)	163.8	368.7	115.0
